# Age-Friendly Care: What Matters Most to Older Adults in Ambulatory Care Clinics

**DOI:** 10.1093/geroni/igaf122.3153

**Published:** 2025-12-31

**Authors:** Sherry Greenberg, Nicholas Schiltz, Grace Armstrong, Anne Pohnert, Mary Dolansky

**Affiliations:** Hunter-Bellevue School of Nursing, New York, New York, United States; Case Western Reserve University, Cleveland, Ohio, United States; Case Western Reserve University, Cleveland, Ohio, United States; CVS Health, Woonsocket, Rhode Island, United States; Case Western Reserve University, Cleveland, Ohio, United States

## Abstract

Considering patient preferences by asking ‘What Matters’ most, key to the Age-Friendly Health Systems 4Ms framework, fosters patient-centered care and improves health outcomes. MinuteClinic, comprised of approximately 1,100 convenient care clinics across 35 states and DC, implemented the 4Ms framework in May 2020 as part of routine care for nearly all older adults. This retrospective cross-sectional study aimed to describe What Matters most to older adults at MinuteClinic locations across the US between January 2021 and March 2024. Data were extracted from electronic health records of those aged 65+ who received 4Ms care and responded to the ‘What Matters’ question, “What matters most to you?” Responses were categorized by providers into predefined categories: social activities/inclusiveness, health, family togetherness, independence, and other. Results demonstrated that 388,046 older adult patients responded to the ‘What Matters’ question. The most common response was engagement in social activities/inclusiveness (49%), then health (21%), independence (17%), and family togetherness (11%). Over time, responses for “social activities/inclusiveness” increased from 38.6% to 54.9%, while “health” decreased from 26.3% to 19.1%, and “family togetherness” decreased from 16.8% to 6.3%. This is the largest reporting of What Matters most to patients in ambulatory care settings. Approximately 80% of patients identified something other than their own health as what matters most to them, noteworthy given MinuteClinic commonly addresses urgent health needs. This study highlights the significance of understanding “What Matters”, revealing that social activities and inclusiveness are priorities, thereby underscoring the need to promote patient-centered care that aligns with values and preferences.

